# Efficacy of Drainage Combining Endoscopic Retrograde Cholangiopancreatography With Endoscopic Ultrasound‐guided Biliary Drainage for Difficult‐to‐Control Cholangitis in Patients With Hilar Cholangiocarcinoma

**DOI:** 10.1002/deo2.70210

**Published:** 2025-09-26

**Authors:** Tomoki Ogata, Yusuke Kurita, Takamitsu Sato, Shin Yagi, Yu Honda, Takeshi Iizuka, Sho Hasegawa, Kunihiro Hosono, Noritoshi Kobayashi, Itaru Endo, Kensuke Kubota, Masato Yoneda, Atsushi Nakajima

**Affiliations:** ^1^ Department of Gastroenterology and Hepatology Yokohama City University Hospital Yokohama Japan; ^2^ Department of Oncology Yokohama City University Hospital Yokohama Japan; ^3^ Department of Gastroenterological Surgery Yokohama City University Hospital Yokohama Japan

**Keywords:** combining endoscopic retrograde cholangiopancreatography (ERCP) with EUS‐BD drainage, difficult‐to‐control cholangitis, endoscopic ultrasound‐guided biliary drainage (EUS‐BD), hilar cholangiocarcinoma, stent patency

## Abstract

**Objectives:**

Hilar cholangiocarcinoma often results in repeated early stent dysfunction and difficult‐to‐control cholangitis after drainage using endoscopic retrograde cholangiopancreatography (ERCP). In this study, we evaluated the effectiveness of additional drainage using endoscopic ultrasound‐guided biliary drainage (EUS‐BD) in patients with hilar cholangiocarcinoma who had difficult‐to‐control cholangitis after transpapillary drainage with ERCP alone.

**Methods:**

We retrospectively evaluated 20 patients with hilar cholangiocarcinoma who had difficult‐to‐control cholangitis after transpapillary drainage with ERCP at our hospital between 2017 and 2025 and therefore underwent additional drainage using EUS‐BD. We evaluated the time to recurrent biliary obstruction (TRBO) just before and after combined ERCP and EUS‐BD in these patients.

**Results:**

The Bismuth classification of stenosis was II in four cases (20.0%), IIIa in five cases (25.0%), IIIb in one case (5.0%), and IV in 10 cases (50.0%). The median (95% confidence interval) TRBO biliary obstruction just before and just after additional drainage with EUS‐BD was 16.5 days (7.0–27.0) and 91.0 days (53.0–NR), respectively, and additional drainage with EUS‐BD significantly prolonged stent patency.

**Conclusions:**

Combining ERCP with EUS‐BD for drainage was effective in patients with hilar cholangiocarcinoma who had stent dysfunction due to cholangitis that was difficult to control using transpapillary drainage with ERCP alone.

## 1 Introduction

Transpapillary stent placement with endoscopic retrograde cholangiopancreatography (ERCP) is the preferred treatment for the palliation of malignant distal biliary obstruction [1, 2, 3]. Hilar cholangiocarcinoma sometimes requires multiple biliary drainage [4], and ERCP alone is often problematic in some cases, with repeated short‐term stent dysfunction and difficulty in controlling cholangitis [5].

Endoscopic ultrasound‐guided biliary drainage (EUS‐BD) was developed as a treatment for biliary obstruction and has gained popularity in recent years, with studies reporting its high technical and clinical success rates [6, 7, 8, 9, 10]. Several studies have reported the usefulness of drainage using EUS‐BD in patients with hilar cholangiocarcinoma [11, 12, 13, 14]; however, the efficacy of drainage combining ERCP with EUS‐BD for hilar cholangiocarcinoma with repeated early stent dysfunction or difficult‐to‐control cholangitis is currently unclear. Therefore, we retrospectively evaluated the efficacy and safety of combining ERCP with EUS‐BD in patients with hilar cholangiocarcinoma who had repeated early stent dysfunction and difficult‐to‐control cholangitis after transpapillary drainage using ERCP.

## 2 Methods

### 2.1 Patients

In total, 160 patients with hilar cholangiocarcinoma were treated at our hospital (Yokohama City University Hospital) between 2017 and 2025. Transpapillary drainage with ERCP was performed as the initial treatment in all patients. At our institution, combining ERCP with EUS‐BD drainage was performed in patients with cholangitis difficult to control by transpapillary drainage with ERCP alone. Indications were patients with stent dysfunction within 30 days. In total, we performed a combination of ERCP and EUS‐BD drainage in 20 cases with hilar cholangiocarcinoma in whom transpapillary drainage with ERCP alone was difficult during the study period. In this study, we retrospectively examine these cases.

This study was approved by the Institutional Review Board of Yokohama City University Hospital (F241200003). In this retrospective observational study, only medical information was used, and there was no invasion of participants’ privacy. All patients received an opt‐out form for informed consent. Patients who did not provide consent were excluded.

### 2.2 EUS‐BD Procedures

In all cases, EUS‐BD was performed by endoscopists skilled in EUS‐guided procedures. Endoscopists skilled in EUS‐guided techniques were defined as having experience with at least 20 cases [8]. In principle, EUS‐BD was performed following transpapillary stent exchange or concurrently. In cases with duodenal stenosis in which transpapillary stent exchange was technically impossible, the exchange was not performed. The standard EUS‐BD procedure at our institution was as follows. The endoscopic ultrasound device (EU‐ME2 and GF‐UCT260; Olympus, Tokyo, Japan) was manipulated from inside the stomach or duodenum to visualize the dilated intrahepatic bile duct (segment 3 [B3], segment 2 [B2] or segment 6 [B6]). When performing EUS‐BD for left lobe drainage, B3 is the first choice for puncture. If puncturing B3 is difficult, B2 is selected as an alternative. For right hepatic drainage, B6 is chosen for puncture. While these principles were followed, bile ducts insufficiently drained by transpapillary stents were prioritized for puncture, aiming to achieve drainage of more than 50% of the total liver volume. A 19 G needle or a 22 G needle (Sono Tip Pro Control; Medi‐Globe GmbH, Rosenheim, Germany; Expect; Boston Scientific, Boston, MA, USA; or EZ shot 3 plus; Olympus, Tokyo, Japan) was then used to puncture the bile duct without intervening blood vessels, using color doppler ultrasonography. After puncturing the bile duct, the bile juice was aspirated, and a contrast was used to confirm the bile duct. A guidewire (0.025‐inch VisiGlide 2 for 19‐G needle; Olympus, Tokyo, Japan or a 0.018‐inch Fielder18 for 22‐G needle; Asahi Intec, Aichi, Japan) was then inserted into the bile duct, and a 7‐Fr bougie dilator catheter (ES Dilator; Zeon Medical Co., Tokyo, Japan) was used for fistula dilation to the bile duct. Subsequently, at the discretion of the performing physician, duct dilatation was performed using a 4‐mm balloon catheter (REN biliary dilatation catheter; KANEKA, Osaka, Japan), or drill dilator (Tornus ES; Asahi Intec, Aichi, Japan), or an endoscopic diathermic dilator (Fine025; Medico's Hirata Inc., Osaka, Japan).

After duct dilation, a self‐expandable metallic stent (SEMS) of 6 mm in diameter and 10 or 12 cm in length (Niti‐S S‐type biliary stent; Taewoong Medical Co., Seoul, Korea; HANAROSTENT Biliary Full Cover Benefit; Boston Scientific, Boston, MA, USA; or EGIS Biliary Full Cover Stent; SB‐KAWASUMI Laboratories, Inc., Kanagawa, Japan) or a plastic stent (PS) of 7 Fr in diameter and 14 cm in length (Through & Pass Type IT; Gadelius Medical, Tokyo, Japan) or 7 Fr in diameter and 12 or 15 cm in length (Flexima Biliary Stent; Boston Scientific Boston, MA, USA) or 5 Fr in diameter and 15 cm in length (Harmo Ray; Hanaco medical Co., Saitama, Japan) were placed. The final selection of the stent was determined at the discretion of the endoscopist based on clinical judgment.

### 2.3 Endpoints

The primary endpoint was the time to recurrent biliary obstruction (TRBO) after additional drainage with EUS‐BD in cases of hilar cholangiocarcinoma. The TRBO was defined as the period between the stent placement and the occurrence of recurrent biliary obstruction (RBO) [15]. The RBO was defined as stent occlusion or migration of either the transpapillary stent or the EUS‐BD stent after achieving technical and clinical success, resulting in RBO or other conditions requiring biliary drainage or stent removal [15]. Patients were evaluated just before and after additional drainage using EUS‐BD to compare stent patency. TRBO just before combining ERCP with EUS‐BD was defined as the time from the last transpapillary drainage with ERCP and endoscopic biliary stenting placement to RBO. TRBO, just after combining ERCP with EUS‐BD, was defined as the time from EUS‐BD stent placement to RBO.

The secondary endpoints were the rates of technical success, clinical success, and adverse events associated with EUS‐BD. Technical success was defined as successful placement of at least one stent or catheter in the intended location of the bile duct. Clinical success was defined as the resolution of cholangitis for cases with cholangitis within 14 days [15]. We defined adverse events associated with EUS‐BD in accordance with the lexicon of the American Society for Gastrointestinal Endoscopy [16].

### 2.4 Statistical Analyses

All statistical analyses were performed using the JMP version 17.0 software (SAS Institute Inc., Cary, North Carolina, USA), with statistical significance set at *p* < 0.05. The TRBO was estimated using the Kaplan‐Meier method, and differences between curves were evaluated using the log‐rank test and Cox proportional‐hazards model. The TRBO just before and just after combining ERCP with EUS‐BD drainage, and the patient survival period were then evaluated using a 95% confidence interval (CI).

## 3 Results

Of the 160 patients who underwent transpapillary drainage with ERCP for hilar cholangiocarcinoma between 2017 and 2025, 20 patients had difficult‐to‐control cholangitis and therefore underwent EUS‐BD.

**FIGURE 1:**
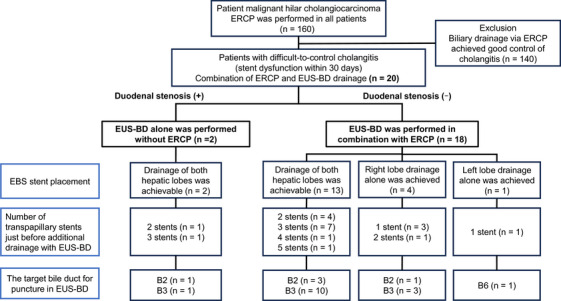
Strategy of biliary drainage in this study. EBS; Endoscopic biliary stenting, ERCP; endoscopic retrograde cholangiopancreatography, EUS‐BD; Endoscopic ultrasound‑guided biliary drainage.

The patient characteristics are shown in Table 1. The Bismuth classification of stenosis was II in four cases (20.0%), IIIa in five cases (25.0%), IIIb in one case (5.0%), and IV in 10 cases (50.0%). The number of transpapillary stents immediately just before additional drainage with EUS‐BD was one in four cases (20.0%), two in six cases (30.0%), three in eight cases (40.0%), four in one case (5.0%), and five in one case (5.0%). In this study, the reason that only one transpapillary stent was placed in some cases was that, in certain patients, the stricture was extremely tight, making selective guidewire insertion difficult, or stent insertion itself was not feasible, which made it impossible to place more than one stent during transpapillary stenting. The median number of stent dysfunctions experienced prior to additional drainage with EUS‐BD was 5.5 (range, 1–21). Moreover, the median duration of stent dysfunction immediately just before additional drainage with EUS‐BD was 16.5 days (95% CI, 7.0–27.0). Figure 1 shows the outcomes of the biliary drainage employed in this study.

**TABLE 1:** Patient characteristics.

No. of cases	*n* = 20
Age (years), median (range)	70 (49–84)
Sex (%)	
Male	13 (65.0)
Female	7 (35.0)
Type of stenosis (Bismuth classification) (%)	
Type II	4 (20.0)
Type IIIa	5 (25.0)
Type IIIb	1 (5.0)
Type IV	10 (50.0)
Number of transpapillary stents just before additional drainage with EUS‐BD (%)	
1	4 (20.0)
2	6 (30.0)
3	8 (40.0)
4	1 (5.0)
5	1 (5.0)
Type of transpapillary stents just before additional drainage with EUS‐BD (%)	
PS	17 (85.0)
PS and SEMS	2 (10.0)
SEMS	1 (5.0)
Number of times of stent dysfunction experienced prior to additional drainage with EUS‐BD, median (range)	5.5 (1–21)
Duration of stent dysfunction just before additional drainage with EUS‐BD (days) (TRBO of only EBS stent), median (95% CI)	16.5 (7.0–27.0)

CI, Confidence interval; EBS, Endoscopic biliary stenting; EUS‐BD, Endoscopic ultrasound‑guided biliary drainage; PS, Plastic stent; SEMS, Self‐expandable metal stent; TRBO, Time to recurrent biliary obstruction.

### 3.1 Outcomes of Combining ERCP With EUS‐BD Drainage

Table 2 shows the outcomes of additional drainage with EUS‐BD. Notably, the technical success rate was 100% (20/20), while the clinical success rate was 90.0% (18/20). Adverse events included peritonitis in two cases; one case was successfully managed with conservative treatment alone, while another developed an abscess due to bile leakage, for which EUS‐guided drainage was performed, resulting in clinical improvement. However, no other serious adverse events were observed.

**TABLE 2:** Outcome of additional drainage with endoscopic ultrasound‑guided biliary drainage (EUS‐BD).

No. of cases	*n* = 20
Procedure time (min), median (range)	30.5 (14.0–145.0)
Bile duct diameter (mm), median (range)	6.1 (4.0–15.0)
Puncture site (%)	
B2	5 (25.0)
B3	14 (70.0)
B6	1 (5.0)
Type of stent at EUS‐BD (%)	
SEMS	8 (40.0)
PS	12 (60.0)
Technical success	100.0% (20/20)
Clinical success	90.0% (18/20)
Adverse events (%)	
Peritonitis	2 (10.0)
Biloma	0
Bleeding	0
TRBO (days), (TRBO of EBS stent and EUS‐BD stent), median (95% CI)	91.0 (53.0–NR)
Patients who were able to continue chemotherapy after the addition of EUS‐BD (%)	10 (50.0)
Patients who underwent surgery after the addition of EUS‐BD (%)	1 (5.0)
Patient survival period just after the addition of EUS‐BD (days), median, (95% CI)	168.5 (107.4–539.8)

CI, Confidence interval; EBS, Endoscopic biliary stenting; EUS‑BD, Endoscopic ultrasound‑guided biliary drainage; PS, Plastic stent; SEMS, Self‐expandable metal stent; TRBO, Time to recurrent biliary obstruction.

**FIGURE 2:**
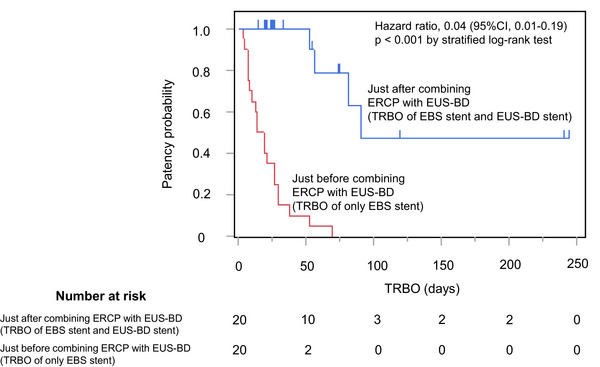
Kaplan‐Meier curves of the TRBO immediately before and after additional drainage using EUS‐BD. The median TRBO just before additional drainage using EUS‐BD (TRBO of only EBS stent) was 16.5 days (95% CI, 7.0–27.0), while that just after additional drainage using EUS‐BD (TRBO of EBS stent and EUS‐BD stent) was 91.0 days (95% CI, 53.0–NR). Furthermore, the TRBO was significantly prolonged with the addition of EUS‐BD (HR, 0.04; 95% CI, 0.01–0.19; *p* < 0.001). CI, Confidence interval; EBS, Endoscopic biliary stenting; EUS‐BD, Endoscopic ultrasound‑guided biliary drainage; HR, Hazard ratio; TRBO, Time to recurrent biliary obstruction.

Figure 2 shows the Kaplan–Meier curves of TRBO before and after additional drainage with EUS‐BD. The median TRBO just after additional drainage using EUS‐BD was 91.0 days (95% CI, 53.0–NR). In contrast, the median TRBO immediately just before additional drainage with EUS‐BD was 16.5 days (95% CI, 7.0–27.0). Notably, the median TRBO was significantly prolonged by additional drainage with EUS‐BD (hazard ratio [HR], 0.04; 95% CI, 0.01–0.19; *p* < 0.001). In this study, RBO was observed in four cases after ERCP and EUS‐BD, and CT revealed that the cause was attributed to the EUS‐BD stent in two cases, the transpapillary stent in one case, and both the EUS‐BD stent and the transpapillary stent in one case. In all four cases, successful replacement of the stent responsible for RBO was achieved, and no patient required percutaneous transhepatic biliary drainage (PTBD). After additional drainage with EUS‐BD, chemotherapy could be administered in 10 cases (50.0%), and surgery could be performed in one case (5.0%). The median survival time after additional drainage using EUS‐BD was 168.5 days (95% CI, 107.4–539.8).

## 4 Discussion

This study retrospectively evaluated the efficacy of additional drainage using EUS‐BD for cholangitis in patients with hilar cholangiocarcinoma, which is often difficult to control using transpapillary drainage with ERCP alone. We found that the median TRBO was significantly prolonged after combined ERCP and EUS‐BD drainage. Thus, our findings show that ERCP combined with EUS‐BD prolongs the TRBO in patients with hilar cholangiocarcinoma and difficult‐to‐control cholangitis. Therefore, this combination of ERCP and EUS‐BD may be useful in such patients, showing high technical and clinical success rates.

Both PS and SEMS were used for EUS‐BD. Various reports have discussed the advantages and limitations of PS and SEMS in EUS‐BD. PS can be delivered using a thin delivery system; however, fistula dilation is often required, which raises concerns regarding bile leakage [17]. In contrast, SEMS are associated with a lower incidence of bile leakage compared with PS, but the risk of segmental cholangitis remains [18]. Therefore, stent selection should be based on anatomical conditions and the risk of adverse events in each case.

In this study, the TRBO was prolonged by performing additional drainage using EUS‐BD in patients with hilar cholangiocarcinoma, which is often difficult to control with transpapillary drainage using ERCP alone. The addition of EUS‐BD allows the drainage of the left bile duct, and this drainage route does not come into contact with the tumor, which may enhance the drainage effect [12]. In this study, before the addition of EUS‐BD to ERCP, cholangitis was difficult to control, and continuous chemotherapy was difficult due to early RBO. However, after additional draining using EUS‐BD, the TRBO was prolonged in 10 cases, which made chemotherapy possible. Additionally, surgery was possible in 1 case. Therefore, the combination of ERCP and EUS‐BD for drainage may make it possible to continue chemotherapy or perform surgery in patients with cholangitis that is difficult to control with transpapillary drainage using ERCP alone, thereby contributing to a prolonged prognosis in those who are difficult to treat continuously.

PTBD is a method of drainage that is used when ERCP is not possible or when transpapillary drainage with ERCP is insufficient. However, in a previous randomized controlled trial comparing endoscopic ultrasound‐guided transmural biliary drainage and PTBD, the PTBD group had a significantly higher rate of adverse events (8.8% vs. 31.2%; *p* = 0.022) [19]. PTBD involves the placement of an external fistula tube, which reduces the patient's quality of life [12]. In contrast, the tubes used for EUS‐BD are endostomies; therefore, no risk of self‐extraction of the tube exists, and drainage is expected to occur without compromising the patient's quality of life. In addition, using this method, the bile juice is absorbed by the body, thereby maintaining enterohepatic circulation, metabolism, and vitamin absorption. This is beneficial for the liver chemistry and immune function [20]. Based on these results, if cholangitis cannot be controlled with ERCP alone, additional EUS‐BD may be safer and more effective than PTBD because it preserves the quality of life and enterohepatic circulation of bile juice.

The present study had some limitations. First, this was a retrospective, single‐center study. Second, this study included a small number of patients. Therefore, although the study demonstrates the efficacy and safety of combined ERCP and EUS‐BD drainage in a small population, future studies are required to confirm these results in a larger population.

In conclusion, combined ERCP and EUS‐BD drainage for hilar cholangiocarcinoma significantly prolongs the duration of stent patency and may be useful for patients with hilar cholangiocarcinoma in whom cholangitis is difficult to control.

## Author Contributions

All authors participated in conducting this research. **Tomoki Ogata**: manuscript writing, drafting conception, and design; **Yusuke Kurita**: drafted, conceived, and designed the study, performed the endoscopic procedures, and assisted with manuscript writing; **Takamitsu Sato**, **Shin Yagi**, **Yu Honda**, **Takeshi Iizuka**, **Sho Hasegawa**, **Kunihiro Hosono**, and **Kensuke Kubota**: performed endoscopic procedures and analyzed the data; **Noritoshi Kobayashi**, **Itaru Endo**, **Masato Yoneda**, and **Atsushi Nakajima**: provided clinical advice. All authors have approved the final draft of the manuscript.

## Ethics Statement

**Approval of the research protocol by an Institutional Review Board**: This study was approved by the Institutional Review Board of Yokohama City University Hospital (F241200003).

## Consent

Not applicable. In this retrospective observational study, only medical information was used, and there was no invasion of participants’ privacy. All patients received an opt‐out form for informed consent. Patients who did not provide consent were excluded.

## Conflicts of Interest

The authors declare no conflicts of interest for this article.

## Data Availability

The datasets used and/or analyzed in the current study are available from the corresponding author upon reasonable request. Not applicable.
